# Clinical and Pathological Reports of Cases of Insanity

**Published:** 1884-05

**Authors:** S. V. Clevenger

**Affiliations:** Special Pathologist Cook County Hospital for the Insane


ARTICLE III.

### CLINICAL AND PATHOLOGICAL REPORTS OF CASES OF INSANITY.

By S. V. CLEVENGER, M.D., Special Pathologist Cook County Hospital for the Insane.

Female case No. 119. Terminal Dementia. Matilda A., age 30; admitted Aug. 31, 1880; Swede; married. Was suicidal and maniacal with religious delusions. Tried to eat her own shoulder, explaining that as Christ died for her she might as well eat herself for Him. Brother insane; father died of phthisis pulmonalis. Has three children; youngest born just before becoming insane; thus her dementia follows puerperal insanity, but as I have noticed in so many cases with the puerperal condition as an exciting cause, the predisposition was hereditary, as indicated by the brother being insane.

It is well to inquire into the family antecedents of the puerperally insane, for hereditary taint seems to predominate in them.

This case also shows how little importance need attach to religious excitement as a cause of insanity. It was in her case a phase of the mental alienation, as in many others regarded as religious lunatics, Political, religious, or any kind of excitement merely, as a rule, precipitates the insanity, and should not be blamed as a cause in those predisposed.

Phthisis in ancestry of insane may be viewed from many points. Statistics in general, so far, throw but little light upon it, as the prevalence of the disease among the parents of sane persons may show no disproportion. From 329 males and 439 females in this asylum it was ascertained that relatives of insane died from this disease as follows: Fathers of 12 males, 9 females; mothers of 5 males, 9 females; both parents of 2 females; 1 brother of a male. Total fathers 23; mothers 16. Total male insane with phthisis in ancestry 17; total females insane with same 20, so far as ascertained, which was in a very small percentage of cases.

Female case No. 147. Epileptic Insanity. Jane N., age 30; admitted Sept. 29, 1881; English; single. Furor when excited, independently of the epilepsy; the latter is often severe; three or four attacks daily with remissions of two to three weeks, and worse at menstrual periods. Acts a little sillily after paroxysms of epilepsy, with slight stupor, from which she emerges apparently sane. Mother died in child-bed; father living and well; epilepsy began when 14 years old at menstrual inception; recently convulsions occurred, upon Dec. 28, 1883, Jan. 3, 1884, Feb. 7, Feb. 9, Feb. 13, Feb. 27, March 18.

Female case No. 146. Hypomania. Honora G., age 19; admitted Sept. 20, 1881; domestic; Irish; single. Always happy, with a little of the silliness of hebephrenia, but usually sits at a table with a book (generally one she cannot understand). Fond of dress; coherent, pleasant; was sun-struck June, 1879. Has a cousin here, also insane, female case No. 424.

Male case No. 82. Larvated Epileptic Insanity. Joseph H., age 22; admitted April 30, 1881; cigarmaker; single. Usually quiet and well-behaved; trusted about grounds; cacophorous at times; vociferates and talks vile nonsense; had hæmorrhoids just before insane, anæmic, but otherwise fairly nourished, pupils dilated, pulse 102, heart's action irregular. Younger brother epileptic. Past two years complained of headache and dizziness; has had several seizures, during which consciousness was lost, preceded by excitement, walking, laughing, and whistling. This is a form of epilepsy wherein the usual convulsions are replaced by a psychic equivalent. An important matter to recognize, as the treatment for ordinary epilepsy is indicated in this form of insanity.

Male case No. 120. Mania. Daniel S., age 54; admitted Aug. 3, 1883; negro; teamster. During maniacal access imagines himself wealthy; owns coal mines; hallucinations of sight and hearing; scar on right forehead; stole two dry goods boxes and contents from sidewalk; arrested and committed, but found to be insane. Once saw God in a chariot at his window, who told him to break out. Patient kicked out panels of door, and armed with one, he demolished the contents of his room, keeping up a fire of everything portable until the room was empty. Attendants then rushed upon him, but it required six men a long while to subdue him, owing to prodigious strength. July, 1883, apparently recovering, but Sept. 5, 1883, severe pneumonia began and ended his life Sept. 15, 1883. Autopsy eight hours after death; aural and temporal muscles large; medulla small in region of olivary bodies; arterial engorgement in isthmus cerebri; also in Rolandic, left region, and in frontal parietal and temporal, but greatest in occipital parts; rusty aspect of dura along superior longitudinal sinus; veins of left side more full than right; stasis right side of cerebellum. Little found besides evidences of former inflammatory lesions, in minor degree, referable to his insanity.

Male case No. 123. Melancholia. F. C. W. A., age 62; admitted Aug. 24, 1882; German; widower; butcher. Senile amnesia for recent events. Large irreducible scrotal hernia; was not intemperate; lost much property and grew despondent; has delusion that he is to be hanged at 10 A. M. of every succeeding day. Says he does not know why he is to be hanged, that he does not deserve it. Visitors are often introduced to him as the State Governor, with a reprieve. He is profuse in his thanks, and for the time lifted from his despondency, but in an hour forgets all about the matter and relapses into the fixed delusion. When argued with, he says that he realizes the insanity of his idea, and that he might be able to overcome it outside the building. Nevertheless, this admission is more of a ruse to obtain his liberty. His hernia renders him quite helpless, and when the tumor is more than ordinarily troublesome, his mental condition is correspondingly worse.

Male case No. 129. Katatonia. Leopold P., age 33; admitted September 7, 1882; German; married; peddler. Cataleptoidal July, 1883; reticent and melancholy following December. No heredity; was sun-struck in July, 1881, confining him to bed awhile, but worked after recovery till summer of 1882. Feels better in cold than in warm weather, which hurts his head. Sunstruck again in summer of 1882. Has had remissions of a few days in each month during which he held short conversations. Melancholia followed first attack, before which he suffered from pain and heated sensation of head January 1, 1884. Emerging from stupor February 8, 1884, attacked attendant with knife which he took from dining room table; is passive now, March, 1884.

Male case No. 136. Chronic Alcoholic Insanity. S. F. S., age 55; admitted October 12, 1882; American; married; hotel keeper. Usually elated and garrulous; talks coherently, but is a great bore with his conceitedness; affects piety and quotes Scripture one minute, and swears the next. Father died with phthisis pulm.; mother, in child-bed. Patient is industrious and thorough in his work, but very excitable; pulse rapid and thin; heart tumultuous. February 21, 1884, threatened night watchman; February 27, 1883, furor, noisy, belligerent; tried to break down door of room; when calmer is quite an efficient nurse for sick insane; his main defects being garrulity and occasional explosions of fury.

Male case No. 137. Larvated Epileptic Insanity. Jas. S., age 20; admitted October 16, 1882; American; single; mania recurs about once in three weeks, lasting a day or two. Destructive; suicidal; homicidal; struck patients without provocation several times; quite rational in interim. When insanity began, noticed to hide from his companions, and just before furor grew pale in face, followed by flushing. At his home every article of furniture shows marks of his violence. He claims a relish for blood. February 8, 1884, noisy and belligerent; growing demented.

Male case No. 145. Epileptic Insanity. Edw. H., age 42; American; single; brakeman. Epilepsy ceased December, 1883, and profound dementia set in; hæmatoma auris slight. Died January 3, 1884; no autopsy.

Female case No. 241. Recurrent Melancholia. Julia C., age 42; admitted April 19, 1883; Irish; single. Parents died aged 73 and 82 years. Patient physically healthy. Apathetic, despondent, prays much, refuses to answer questions. Former attacks: 1. Twenty-five years ago; lasted eight months. 2. Sixteen years ago; lasted six months. 3. Two years ago; lasted two months. 4. Began February, 1883; lasted eight months. Menses regular; not painful till last attack, which began with dysmenorrhœa. Discharged Dec. 6, 1883. Friends say her last recovery is the most decided, which is not an unusual circumstance in such cases after the menopause.

Female case No. 234. Melancholia. Catherine B., age 45; admitted April 12, 1883; Bohemian; married. Brother was here insane; now at home demented; nephew also was here insane. Husband a drunkard; had much domestic trouble; her daughter died and patient was caring for her children, became pyromaniacal. Relatives wrote to Prague and had ancestry traced back three generations and could learn of no insanity among them. They think too much beer had something to do with it, but change of environment appears to be main cause. Ancestry had been well-to-do farmers, living monotonous, quiet lives, and descendants were not accustomed to the rush and bewilderment of such large cities as Chicago. "As soon as anything went wrong they seemed to go insane." The brother had built a house beyond his means, failed and lost his mind.

Male case No. 198. Paretic Dementia. Randall W., age 28; admitted April 12, 1883; American; single. Deaf, partially blind; other members of family deaf; was three years at Newsboys' Home, and two years at Home of Incurables, with locomotor ataxia, and delusions of persecution gradually appeared during residence at latter place.

An interesting feature in this case is the ataxia preceding the paresis, some alienists claiming that paretic dementia is a form of cerebral ataxia allied closely to the locomotor.

Male case No. 199. Paranoia. Thos. K., age 26; admitted May 17, 1883; Irish; single; laborer. Three months insane before admission; parents old and feeble but sane; patient used to hang around churches; had the name of a girl "called" in banns in church without her knowledge; made constant efforts at escape; talks rationally, but argues on subject of religion and is erotic; claims he must marry every female he sees. Gradually improved mentally and grew fat. Discharged apparently recovered March 21, 1884. When a patient improves physically as indicated by acquired fleshiness, and his mentality does not improve at the same time, it augurs ill for the patient, but both body and mind together improving, the chances are more favorable than where mentality returns without restitution of bodily vigor.

Male case No. 204. Paranoia. Terrence M., age 55; admitted April 19, 1883; Irish; single; laborer; was in several wars in English army; tidy; appears intelligent, but refuses food till forced to eat by hunger. Insanity began with melancholic depression; thought he was to be hanged. Exhibits the interesting form of paranoia known among alienists as *mysophobia* or fear of contamination. His aversion to eating with others arises from the idea that he debases himself by so doing. Refuses to go to bed from similar motives; troublesome and beligerent at times; talks incessantly on religious subjects; says his "spirit and soul were burned up in the stove; that the others in ward are all robbers, and contact with them ruins him. Makes a circuit of ward looking for his soul.

Male case No. 205. Mania from Traumatism. John G., age 45; admitted April 26, 1883; American; married; machinist. Irritable; homicidal; suicidal; destructive; noisy; suspicious; erotic; jealous of wife. Nothing ascertained of ancestry. Was struck by lightning in 1880; suffered mainly pain in head and stomach, but was conscious and talked rationally until the third day, when left hemiplegia supervened, lasting nearly a year. The paralysis was complete, including facial, with ptosis. Tongue and vocal organs first to recover, in 1881, when insanity became evident. Lightning showed marks along forehead, neck, and left side. Has become quieter since admission, tending toward dementia.

Male case No. 215. Syphilitic Insanity. Jas. O., age 21; admitted May 17, 1883; American Celt; single. Erotic and religious delusions. Railroad accident crushed off right leg below knee when 9 years old. Father rheumatic; mother illy-balanced. Mother stated that when patient had leg amputated he inhaled so much chloroform that ten years afterward he became crazy. She "always knew the chloroform would come out of him sometime!" It was not ascertained until December, 1883, that patient had been syphilitic; brother said that patient had been treated by homœopaths in July, 1879, for this trouble; that his hair fell out, but the "disease was cured." As soon as this information was received, he was placed upon antisyphilitic treatment. In February, 1884, periostitis appeared, but is mentally improving.

The necessity for strict truth being communicated to physicians, in such cases especially, is evident from the time lost here.

This patient might have been restored to his family sane long ago had the information been given earlier. This form with psychoses arising from rheumatism are the most curable of all. The question naturally arises, why not institute treatment applicable to such cases (kali iodidum, for instance), in all cases where the ætiology was obscure, in the hope of hitting upon the right cause? This would be unjustifiable in many instances, as great harm could easily supervene from such a method. It would be as little justified as in ordinary practice outside of asylums, even though advocated by one author.

Male case No. 216. Senile Dementia. B. H., age 69; admitted May 8, 1883; American; married; mercantile. Amnesic for recent events; passive; polite; effusive; verbigerative; coherent; dwells much upon his early life. Occasional attacks of transitory furor. Cerebral anæmia, with feeble heart action. Maternal uncle insane at age of 30, when he died after a love disappointment. Patient was always eccentric and tyrannical. Constantly regrets the past; ill-tempered, but remorseful; was arrested for assaulting his landlady when she asked him a simple question. Hair has been gray since age of 28. Always temperate. His peculiarities took a more disagreeable turn in 1881. Has dread of poverty and his family suffering; wishes to seek employment when no occasion for it; family very well circumstanced. July 15, 1883, excited at night—thought he was on a steamboat; wanted it to land and let him off; next day fancied he was dead, and lay quietly in bed all day. Memory weakening. Constantly whines abont his being a miserable old creature, though often roused to bursts of fury, during which he is as vituperative as he was before polite. Swears like a pirate at these times, though religious ordinarily. Discriminates in favor of asylum officials, to whom he is always very deferential; unloads his anger upon those he considers of less consequence.

Male case No. 219. Recurrent Melancholia. Wm. L. C., age 50; admitted May 24, 1883; Irish; married; printer. Had been in an asylum before, but was working at his trade when violent bregmatic headache siezed him, with delusions of persecution, and undue desire to shoot a reporter. Gradually recovered and was discharged Feb. 11, 1884.

Male case No. 222. Imbecility. Victor F., age 33; admitted May 29, 1883; Swede; single. Grew worse after great Chicago fire; thought black dog wanted to bite him and witches were after him. Mother is also an imbecile with a venomous tongue and full of phrenological nonsense. Patient spends his entire time prancing up and down ward, slapping his head, as he says, to keep down bad thoughts. Father died of phthisis pulm. Patient is much better off when at work outside, but his mother raises so many objections, and is so offensive with her accusations against the commissioners and asylum officials generally for "killing her son with work" that he is kept from doing anything, to pacify her. It would be well if a law compelled asylum patients to work to the extent of their abilities, as uniformly the world over this is found to be of great assistance to the disordered mind. As it is, where objections are raised by ignorant friends, idleness with its evil effects is enforced.

Male case No. 241. Katatonia. Joseph H., age 39; admitted June 28, 1883; Bohemian; married; blacksmith. Delusions of persecution: often 3 days cataleptoidal, followed by melancholia. In asylum is four or five days cataleptoidal. Was here 4 years ago for 7 weeks; then had delusions of persecution and some katatoniac symptoms. Complains of head paining at night; sees the devil; works well during remissions. Has eight children, all of whom are scrofulous (Kiernan noted scrofulous association in this form of insanity while he was in Ward's Island Asylum, New York). Father and two sisters insane; no furor has appeared in his case, as is usual with katatoniacs. In this form of insanity remissions occur which last very long, but unless appropriate treatment is instituted at the outset chronicity follows. The cataleptoidal stage may be instantly broken up by inhalation of amyl nitrite and with advantage to the patient. Atonicity of the bowels accompanies this disorder.

Male case No. 252. Circular Insanity. Henry W., age 24; admitted July 12, 1883; American-German; single; printer; 13 weeks insane before admission. Melancholic delusions of persecution; suspicious; sullen; dreads personal danger; mostly amnesic. Had pneumonia and small-pox and was never very healthy. Mother died of "heart disease." His melancholia came on suddenly Dec. 28, 1883; severe furor; truly maniacal; not the raptus of melancholia; after breakfast struck theatrical attitudes and gave vent to much stage talk; Jan., 1884, still excited and verbigerative; complete change from former sullenness, which suddenly reappeared Feb. 12, 1884. Theatrical behavior, with the melancholia and mania, cause this case to appear suspiciously like katatonia, but in the absence of the cataleptoidal stages, I have placed him tentatively among circular insane, until the waxy mobility becomes apparent, if it ever does.

Male case No. 259. Stuporous Insanity. Unknown man, age 20; admitted July 16, 1883; American; single; laborer. When admitted, apathetic; unconsciously stuporose; would not speak; walked automatically; nothing disturbed him. Began to improve in December, 1883, and said his name was Johnson, afterwards said it was Sheehan. March, 1884, recovered, but is evidently not overburdened with brains; acts silly, but natural. When asked to go to work, replied: "Catch me working on a county farm."

Male case No. 261. Melancholia. Paul G., age 22; admitted July 26, 1883; German; draughtsman; single. Hypochondriacal; attempted suicide; said he had broken a testicle, and it was running down his leg; jumped into Lake Michigan; was always reticent, and lately drank hard. Recovered rapidly, and was discharged Aug. 10, 1883.

Male case No. 265. Mania. Michael H., age 23; admitted Aug. 2, 1883; American-Celtic; single; gasfitter. Insane five years before admission, following typhoid fever, and gradually growing worse; complete left hemiplegia after fever. Leg recovered activity November, 1863; has had in left eye cataract and orchitis. Homicidal; suicidal; destructive; delusions of persecution; mischievous and treacherous. Mother died of "heart disease."

Male case No. 275. Mania. Andrew V., age 54; admitted Aug. 16, 1883; German; married; expressman. Expansive delusions. "Can kick higher, is stronger and richer than any one else;" tongue coated; pulse rapid; thready. "The world belongs to him;" continuous excitement, with hallucinations of sight and hearing; usually erotic. November, quieter, but delusions persist; improved thereafter rapidly and discharged apparently recovered Dec. 23, 1883. Readmitted April 7, 1884. Had been drinking and was too exhuberent at home.

Male case No. 278. Mania. Lewis A. N., age 26; admitted Aug. 16, 1883; Swede; single; painter; intemperate. Delusions of persecution; imagines people follow him; talks to himself; continually abuses invisible enemies; scar on throat from attempted suicide. Furor Feb. 7 and 12; thought Prussians were after him; would yell: "Hurry up the ammunition wagon!" Feb. 14, stands reflectively in one spot and then spins round three or four times. Feb. 17, excited; lies upon floor and then springs up suddenly as though in battle. Feb. 21, sudden furor; he and another lunatic nearly killed attendant on Ward A1, but prevented through timely rescue by clinical clerk Wm. Cunningham. Patient appeared silly and reeled about ward as though intoxicated in the morning, and dropped dead suddenly March 1, 1884, noon, with face purple from congestion. Autopsy 7 P. M.; scalp thick, fatty and full of blood; leptomeninges bluish discoloration left side apex temporal lobe and in medullary fossa; cerebrum anæmic; extraordinary quantity of cerebro spinal fluid. Numerous small extravasations front of pons and medulla; heart weight, 12½ ounces; no lesions found, though relatives stated he suffered with heart disease; probably angina pectoris.

Male case No. 141. Paretic Dementia. Jas. L., age 41; admitted Oct. 26, 1882; American Celtic; policeman; married; delusions of grandeur and persecution; "Feels first-rate" (the usual assertion in this disease); tremulous speech; was at battle of Ball's Mountain during late war and was shot in sternum, ball lodging in chest; also wounded in back. Father died paralytic, age 65; mother, hæmoptysis, age 58. Mental trouble began in January, 1882, and by January, 1884, memory bad and epileptiform tonic convulsions; Feb. 7, 1884, abed; says he cannot get enough to eat and complains of the taste of his food; Feb. 18, broke window during furor; Feb. 21, says his lungs and other insides are gone and that he only weighs two pounds, and that flour is a million dollars a barrel; thinks everyone in Chicago is starving. These delusions are based upon his general anæsthesia, which is extreme. Sometimes claims he is dead. Feb. 26 and 28, 1884, furor; March 11, 1884, is having furibund attacks twice weekly. A remission lasted from June 24, 1883, to Aug. 4, 1883, at which time he returned to asylum voluntarily, claiming that Dr. Thuemmler's medicine was the only thing that benefited him. (Occasional secale cornutum.)

Male case No. 263. Epileptic Insanity. Phineas Y., age 44; admitted August 2, 1883; German; single; carpenter. Was at Infirmary four years. After fits, stupid and then maniacal. Stabbed a man in infirmary. Attacks occurred usually monthly or second month, often at night. One of the most dangerous lunatics in the asylum. February 27, 1884, knocked a patient down with a chair. He had a deep depression over vertex from an old wound, through which the brain pulsations could be felt. Was quite an adept at card-playing, and would pass for an ordinarily sane man until approach of epilepsy. No particular motor or sensory disturbance noted aside from his usual convulsions. Spoke connectedly, and no mental faculty seemed to be below the standard usual to a person of his class. General anasarca progressed to his death, March 9, 1884. Autopsy next day. Wound one inch square a little to right of median line, over Rolandic sulcus, though skull closed by proliferation of dura mater. Steatomatous tumor an inch in diameter resting on left orbital plate, obliterating left olfactory nerve completely, extending upward and slightly forward two inches, having destroyed the lenticular nucleus and half of the corpus striatum of that side. Lungs œdematous; right lower lobe congested; left lower lobe slightly congested. Anasarca general. Heart had undergone nearly complete fatty degeneration. Each kidney lobulated (arrested fœtal peculiarity). Pectoralis major muscles divided into strands similar to those found in felidæ.

Male case No. 72. Senile Dementia. John W., age 73; admitted Dec. 12, 1880; English; married. Had been deteriorating mentally ten years. Delusions of persecution; hallucinations of hearing, and unsystematized delusions generally. Health had been fair until after admission. Father died aged; mother of cancer. Had a scientific, inventive turn of mind, but with no practical systematic training in matters of education. Was always crotchety; often opposed meat, tea, or salt as a diet for family; was always a vegetarian. His family yielded to his whims until they became unbearable. Was formerly very active; now lies abed all day, thinking up wonderful inventions. At the time the telephone appeared, was greatly excited over it. Has six children, all healthy and sane. No heredity. Died Oct. 16, 1883.

It is noticeable that many of the so-called "peculiar," "queer," "crotchety," "irritable" individuals who manage to reach advanced life, fall into senile dementia, through age apparently exaggerating preëxisting defects in their characters.

In this day, when knowledge is so readily attainable, there is certainly a screw loose in the composition of those "perpetual motion" seekers and other illy-balanced speculative minds constantly drifting off into spiritualism, chiromancy, etc., or who, with the advent of age or sickness, are precipitated into easily recognized mental decrepitude.

Female case No. 155. Melancholia. Lotta R., age 40; American; widow; admitted Feb. 16, 1882. Was evidently a well educated and refined lady, sang excellently, and used choice language, but gradually deteriorated mentally. Very little information concerning her past could be obtained. The clothing brought with her indicated previous good circumstances. Toward the last she shrieked piteously at night, and called for "Charlie," day after day would cry to him from the window: "Come and help me; I cannot stand it; the house is falling in. Help me or I shall go crazy!" These were the "raptus" stages of agitated melancholia.

Autopsy twelve hours after death, which occurred August 23, 1883. Conjunctivæ pale. Rigor mortis slight. No pacchionian granulations. Dura thicker than normal; no adhesions; weight of entire brain only 33 ounces. The atrophy was marked, and especially so in the frontal region, where fluid replaced the cerebral tissue which had retracted from the dura. Patient had frequently claimed that the back part of her brain was gone, an indication of aberrant sensations.

It is of prime importance to listen to the delusions of patients as they narrate them; for example, one who complains that he has no gullet or stomach, indicates thereby the anæsthesia of such parts, and affords a possible clue to the centric seat of disorder, in such a case pneumogastric.

